# Ramucirumab in Combination with Pembrolizumab in Treatment-Naïve Advanced Gastric or GEJ Adenocarcinoma: Safety and Antitumor Activity from the Phase 1a/b JVDF Trial

**DOI:** 10.3390/cancers12102985

**Published:** 2020-10-15

**Authors:** Ian Chau, Nicolas Penel, Andres O. Soriano, Hendrik-Tobias Arkenau, Jennifer Cultrera, Rafael Santana-Davila, Emiliano Calvo, Christophe Le Tourneau, Lars Zender, Johanna C. Bendell, Gu Mi, Ling Gao, Samuel Clark McNeely, Joana M. Oliveira, David Ferry, Roy S. Herbst, Charles S. Fuchs

**Affiliations:** 1 Gastrointestinal Units, Royal Marsden Hospital, London SM2 5PT, UK; 2 General Oncology Department, Medical Oncology Department, Oscar Lambret Cancer Center, 59020 Lille, France; n-penel@o-lambret.fr; 3 Sarah Cannon Research Institute/Florida Cancer Specialists, Englewood, FL 34223, USA; asoriano@flcancer.com; 4 Sarah Cannon Research Institute United Kingdom and University College London, London W1G 6AD, UK; Tobias.Arkenau@HCAHealthcare.co.uk; 5 Sarah Cannon Research Institute/Florida Cancer Specialists Leesburg South, Leesburg, FL 34748, USA; JCultrera@flcancer.com; 6 Department of Medicine, University of Washington Medicine, Seattle Cancer Care Alliance, Seattle, WA 98109, USA; RSantana@seattlecca.org; 7 Early Phase Clinical Drug Development Program, START Madrid-CIOCC, Centro Integral Oncológico Clara Campal, 28050 Madrid, Spain; ECalvo@hmhospitales.com; 8 Department of Drug Development and Innovation, Institut Curie, 75005 Paris, France; Christophe.LeTourneau@curie.fr; 9 Department of Internal Medicine VIII, University Hospital Tübingen, 72076 Tübingen, Germany; Lars.Zender@med.uni-tuebingen.de; 10 Sarah Cannon Research Institute/Tennessee Oncology, Nashville, TN 37203, USA; JBendell@tnonc.com; 11 Eli Lilly and Company, Indianapolis, IN 46285, USA; Mi_Gu@lilly.com (G.M.); LingGao418@gmail.com (L.G.); McNeely_Samuel_Clark@lilly.com (S.C.M.); 12 Eli Lilly and Company, New York, NY 10016, USA; Oliveira_Joana@lilly.com (J.M.O.); Ferry_David@lilly.com (D.F.); 13 Yale Cancer Center, New Haven, CT 06520, USA; Roy.Herbst@yale.edu (R.S.H.); Charles.Fuchs@yale.edu (C.S.F.); 14 Smilow Cancer Hospital, New Haven, CT 06473, USA

**Keywords:** gastric/gastroesophageal junction adenocarcinoma, pembrolizumab, phase 1b, ramucirumab

## Abstract

**Simple Summary:**

The prognosis for gastric cancer remains poor, with a median overall survival of approximately 1 year. Ramucirumab and pembrolizumab have each demonstrated antitumor activity and a favorable safety profile as treatments for patients with advanced gastric/gastroesophageal junction (G/GEJ) cancer in the second and third-line setting respectively. However, both agents failed to demonstrate survival benefit over chemotherapy in the first-line setting. Twenty-eight treatment-naïve patients with advanced/metastatic G/GEJ adenocarcinoma were treated with ramucirumab plus pembrolizumab in this phase 1a/b trial. Our results showed that this combination was well tolerated with no unexpected toxicities, and promising durable survival results, particularly among patients with PD-ligand 1 positive tumors. The results of our study therefore support modulating the tumor microenvironment with dual inhibition of VEGFR2 and PD-1 pathways in the first-line patients with advanced G/GEJ cancer.

**Abstract:**

Ramucirumab (anti-VEGFR2) plus pembrolizumab (anti-PD1) demonstrated promising antitumor activity and tolerability among patients with previously treated advanced cancers, supporting growing evidence that combination therapies modulating the tumor microenvironment may expand the spectrum of patients who respond to checkpoint inhibitors. Here we present the results of this combination in first-line patients with metastatic G/GEJ cancer. Twenty-eight patients (≥18 years) with no prior systemic chemotherapy in the advanced/metastatic setting received ramucirumab (8 mg/kg days 1 and 8) plus pembrolizumab (200 mg day 1) every 3 weeks as part of JVDF phase 1a/b study. The primary endpoint was safety. Secondary endpoints included progression-free survival (PFS), objective response rate (ORR), and overall survival (OS). Tumors were PD-L1-positive (combined positive score ≥ 1) in 19 and -negative in 6 patients. Eighteen patients experienced grade 3 treatment-related adverse events, most commonly hypertension (14%) and elevated alanine/aspartate aminotransferase (11% each), with no grade 4 or 5 reported. The ORR was 25% (PD-L1-positive, 32%; PD-L1-negative, 17%) with duration of response not reached. PFS was 5.6 months (PD-L1-positive, 8.6 months; PD-L1-negative, 4.3 months), and OS 14.6 months (PD-L1-positive, 17.3 months; PD-L1-negative, 11.3 months). Acknowledging study design limitations, ramucirumab plus pembrolizumab had encouraging durable clinical activity with no unexpected toxicities in treatment-naïve biomarker-unselected metastatic G/GEJ cancer, and improved outcomes in patients with PD-L1-positive tumors.

## 1. Introduction

Gastric cancer is the fifth most common malignancy and the third leading cause of cancer mortality globally [1]. Platinum and fluoropyrimidine-based regimens are used worldwide for the first-line treatment of advanced incurable gastric or gastroesophageal junction (G/GEJ) cancer, and have been shown to significantly improve overall survival (OS) compared with best supportive care, but prognosis is still poor [2,3,4]. The addition of epirubicin or docetaxel to platinum and fluoropyrimidine-based regimens may be considered in selected patients [3,4]. Treatment with trastuzumab improves survival for approximately 15–20% of patients who have human epidermal growth factor receptor 2 (HER2) over-expressing G/GEJ cancer [2,5].

For patients with HER2-negative G/GEJ cancers, there have been multiple failures in the first-line setting to improve survival with the addition of a new agent to chemotherapy based regimens, including: the anti-epidermal growth factor receptor antibodies cetuximab [6] and panitumumab [7]; vascular endothelial growth factor (VEGF) pathway targeting antibodies ramucirumab [8,9] and bevacizumab [10]; antibodies targeting the MET receptor ligand rilotumumab [11] and onartuzumab [12]; and the antibody targeting matrix metalloproteinase-9 andecaliximab [13]. More recently, the addition of the checkpoint inhibitor pembrolizumab (an inhibitor of programmed death [PD]-1 protein) to chemotherapy, in patients with a PD-ligand 1 (L1) combined positive score (CPS) ≥ 1 (PD-L1, per 22C3 assay), also failed to improve OS compared with chemotherapy alone, while the monotherapy treatment arm reported non-inferiority for OS [14].

The PD-1/PD-L1 signaling pathway is often hyperactivated in the tumor microenvironment as a mechanism to evade cytotoxic T cell (CD8+) mediated cell death, with upregulation of the immune checkpoint protein PD-L1 on immune and tumor cells playing a crucial immunosuppressive role. This, along with an enhanced immunosuppressive regulatory T cell population, ultimately leads to the downregulation of the cytotoxic response through the dysfunction of CD8+ T cells that express PD-1 [15]. The expression of PD-L1 protein on the surface of tumor and immune cells, indicative of a potentially inflamed but immune checkpoint-restrained tumor microenvironment, has been shown to be predictive of response to anti PD-1/PD-L1 therapies in some tumor settings [15,16]. Additionally, multigene signatures comprised of IFNγ-inducible and other inflammatory genes meant to capture the complexities of the tumor microenvironment beyond PD-L1 protein expression were associated with response to checkpoint inhibitors in some tumor types [17,18,19]. Despite this, increasing evidence shows that an inflamed or PD-L1 positive tumor microenvironment is not always sufficient to achieve clinical benefit from immune therapies targeting the PD-L1/PD-1 axis, and combination strategies may be needed to expand the spectrum of patients who respond to them. 

In a phase 2 study of G/GEJ cancer patients who received ≥ 2 lines of prior therapy, targeting the PD-1 receptor with pembrolizumab benefited a minority of patients, with approximately 80% of patients rapidly progressing, and a response rate of 15.5% in patients with PD-L1-positive tumors (CPS ≥ 1; per 22C3 assay) [16]. This led to the regulatory approval of pembrolizumab monotherapy in this population [16]. In the subsequent phase 3 s-line trial, median progression-free survival (PFS) in PD-L1-positive patients treated with pembrolizumab or paclitaxel was 1.5 months and 4.1 months, respectively, with patients experiencing no survival benefit [20]. Similarly, in the first-line setting, over 60% of patients with PD-L1-positive G/GEJ cancer who received pembrolizumab monotherapy progressed rapidly, with a median PFS of 2.0 months compared with 6.4 months for patients who received chemotherapy, and no survival benefit despite a proportion of these patients sequenced onto second- or third-line treatment [14].

Ramucirumab is approved as single agent or combined with paclitaxel as second-line treatment for advanced/metastatic gastric cancer [21,22]. Targeting the VEGF/VEGF receptor 2 (VEGFR2) pathway may improve the efficacy of checkpoint inhibitors by reprogramming the tumor microenvironment to be more immunosupportive or inflammatory [23]. In a translational trial of metastatic renal cell carcinoma, bevacizumab treatment alone increased the trafficking of CD8+ T cells into the tumor, and the expression of immune related genes and cell markers, such as the major histocompatibility complex and PD-L1 [24]. Further combination treatment with atezolizumab antibody targeting PD-L1 resulted in increased clinical response over that previously obtained with either monotherapy [24]. Targeting VEGFR2 with ramucirumab increased PD-L1 expression and CD8+ T cell infiltration in the tumor microenvironment of patients with advanced gastric cancer and reduced the frequency of immunosuppressive effector regulatory T cells in tumor-infiltrating lymphocytes [25]. Some results from this multicohort trial have been previously reported [26]. In pretreated patients, ramucirumab in combination with pembrolizumab was associated with promising survival and prolonged PFS for patients with PD-L1-positive tumors (per an investigational version of the 22C3 assay) compared with patients with PD-L1-negative tumors in G/GEJ cancer, non-small-cell lung cancer, or urothelial carcinoma [26]. Here, we report the results from the first-line cohort (Cohort A2) of patients with advanced or metastatic G/GEJ cancer and PD-L1-positive or –negative tumors who were treated with ramucirumab plus pembrolizumab. 

## 2. Results

### 2.1. Patients

Between 28 June 2016 and 3 March 2017, 29 patients were enrolled at 11 medical centers/hospitals in the USA, UK, France, Spain and Germany, of whom 28 received study treatment (Figure S1). The median age was 63 years, and all except 1 patient had metastatic disease at baseline. All patients were negative for HER2 protein. Nineteen patients (68%) had PD-L1-positive tumors (10 were CPS ≥ 10), and 6 (21%) had PD-L1-negative tumors (data unavailable for 3 patients) (Table 1). At data cutoff, 3 patients were still receiving treatment, 2 of whom had completed 35 cycles of treatment (approximately 2 years). Median treatment duration for ramucirumab and pembrolizumab was 4.5 months (Table S1). Median relative dose intensity was also similar for ramucirumab and pembrolizumab: 94% and 98%, respectively (Table S1). Ramucirumab dose reductions occurred in 2 (7%) patients, while 19 (68%) patients experienced dose delays. Eleven (39%) patients experienced pembrolizumab dose delays (dose reductions were not permitted).

### 2.2. Safety

All 28 patients experienced ≥ 1 treatment-emergent AE (Table S2) and 27 patients (96%) experienced ≥ 1 treatment-related AE (TRAE). The most common any grade TRAEs were fatigue (*n* = 11; 39%), hypertension (*n* = 8; 28%), and rash (*n* = 7; 25%). These were predominantly grade 1 or 2 (Table 2). Eighteen patients (64%) reported grade 3 TRAEs, most commonly hypertension (*n* = 4; 14%) and elevated alanine/aspartate aminotransferase (*n* = 3; 11% each) (Table 2). No grade 4 or 5 TRAEs were reported. 

Irrespective of treatment relatedness, the most common AE of special interest based on the known safety profiles of ramucirumab (AESI) and pembrolizumab (immune-related AE [irAE]) included: bleeding/haemorrhage events (any grade = 43%, grade ≥ 3 = 14%) and hypertension (any grade = 39%, grade ≥ 3 = 25%) for ramucirumab; gastrointestinal (any grade = 43%, grade ≥ 3 = 11%) and skin adverse events (any grade = 25%; grade ≥ 3 = 4%) for pembrolizumab. All AESI/irAE occurred at grade 3 or lower severity, of which a higher percentage of hypertension AESI and hepatic irAEs (AST/ALT increase) were of grade 3 severity. AESI/irAEs by preferred term are reported in Table 2. Serious AEs (SAEs) occurred in 16 (57%) patients, of which 11 (39%) were treatment-related SAEs (Table S3). Discontinuation due to AE occurred in 3 (10%) patients; 1 patient had grade 2 hypophysitis and 1 patient had grade 3 cholecystitis (SAE), both deemed related to study treatment. The third patient had grade 3 angina pectoris (SAE), not deemed related. Death occurred for 17 (61%) patients, all after treatment discontinuation (16 occurred ≥ 30 days post-discontinuation): 16 due to study disease and 1 due to an SAE of weight loss deemed unrelated to treatment.

Thirteen of 28 (47%) patients received post-discontinuation treatment (PDT) (Table S4). Pharmacokinetics for ramucirumab are shown in Table S5 and Table S6. 

### 2.3. Response and Survival

**Figure 1:**
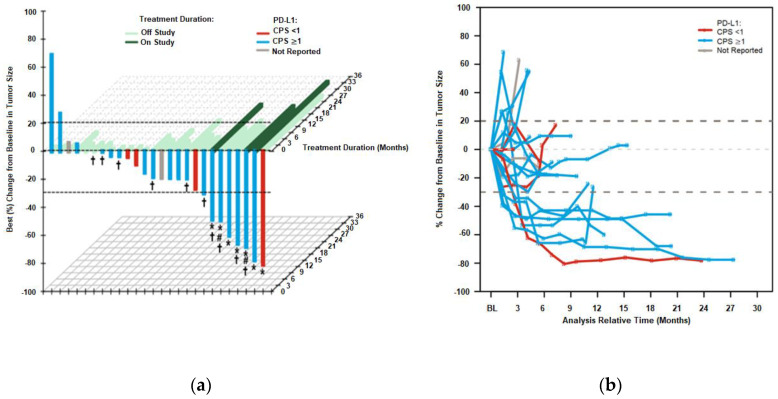
(**a**) Best percentage change of targeted lesions from baseline versus treatment duration. Patients (*x*-axis) are arranged by percentage change in size of targeted lesions from baseline (*y*-axis) and color-coded for best response according to PD-L1 expression. Treatment duration (green) is shown on the *z*-axis. Dotted lines indicate RECIST boundaries (20% to 30%); (**b**) Change in tumor burden by PD-L1 status over time. PD-L1-positive is equivalent to CPS ≥ 1 and PD-L1-negative is equivalent to CPS < 1. Abbreviations: BL, baseline; CPS, combined positive score; PD-L1, programmed death-ligand 1. * Confirmed response. # Microsatellite instability high. † CPS ≥ 10.

Confirmed objective responses occurred in 7 of 28 patients (ORR: 25%), with 1 CR (4%), 6 PRs (21%), and a time to response of 2.7 months. Six of these 7 patients had PD-L1-positive tumors at baseline (CPS ≥ 1), 4 of which were CPS ≥ 10 (Table 3). Two patients had MSI-high tumor samples, while 4 had microsatellite stable (MSS) samples and 1 was not available (Figure 1). The ORR for the PD-L1-positive tumors was 32%, and 40% for CPS ≥ 10 tumors, and was numerically higher than what was observed for PD-L1-negative tumors (17%). At data cutoff, median DOR had not yet been reached, with a lower confidence limit of 9.7 months (Table 3). DCR was similar in patients with PD-L1-negative (67%) and -positive tumors (68%), and higher in the subset with CPS ≥ 10 (80%) (Table 3). The greatest reduction in tumor size was achieved in patients with PD-L1-positive tumors who received the longest duration of treatment and exhibited longer disease control (Figure 1).

**Figure 2:**
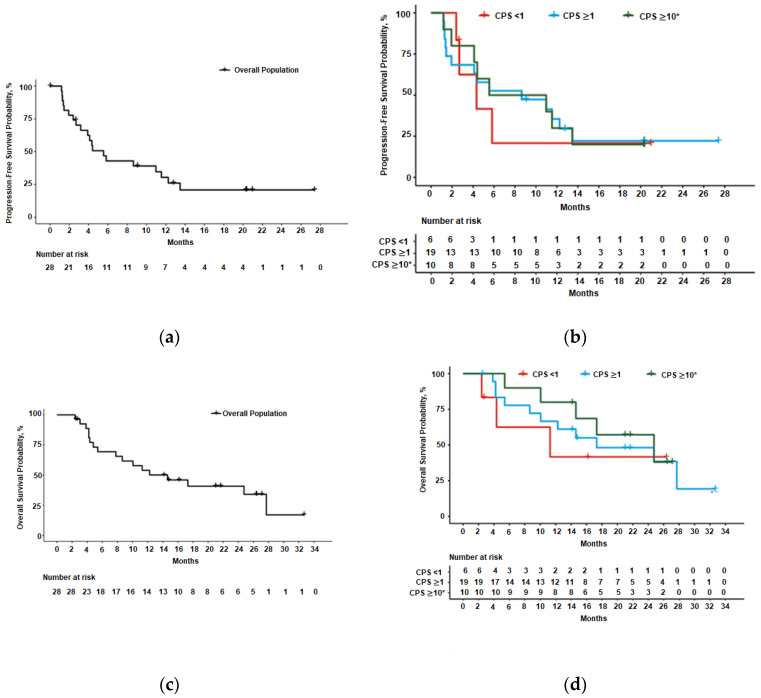
Kaplan-Meier curves of progression-free survival for all patients (**a**) and by PD-L1 status (**b**), and overall survival for all patients (**c**) and by PD-L1 status (**d**). PD-L1-positive is equivalent to CPS ≥ 1 and PD-L1-negative is equivalent to CPS < 1; CPS ≥ 10 is a subset of CPS ≥ 1. Abbreviations: CPS, combined positive score; PD-L1, programmed death-ligand 1.

Median PFS and OS were 5.6 months (95% confidence interval [CI] 2.7–11.5) and 14.6 months (95% CI 5.4–27.7), respectively, in all patients. Patients with PD-L1-positive tumors had numerically higher median PFS compared to PD-L1-negative tumors (8.6 vs. 4.3 months), and median OS (17.3 vs. 11.3 months) (Table 3; Figure 2). Patients with PD-L1 CPS ≥ 10 tumors had a median OS of 24.7 months (Table 3). Twelve-month OS rates were numerically higher in the subset of patients with PD-L1-positive tumors and CPS ≥ 10 compared to PD-L1-negative (66.7% vs. 80.0% vs. 41.7%, respectively) (Table 3). Median OS follow-up was 26.4 months (95% CI 14.8–27.1).

### 2.4. Immune Profiling Analysis

The expression of 3 immune-related gene signatures and associated inflammatory genes was examined in 10 patient tumor RNA samples. The expression of the PD-L1 gene correlated with T cell-inflamed and T-effector gene signatures expression in this subset of patients (Figure S2). The z-score for the 3 gene signatures and the expression of additional genes involved in IFNγ signaling, antigen presentation, T cell activation and recruitment (chemokines/cytokines) appeared elevated in 3 patients who achieved stable disease and in 2 patients with partial responses (Figure S2). Although a trend was observed suggesting gene signatures profiling score was higher in responders versus non-responders (per RECIST), particularly with the T-cell effector signature, these associations did not reach statistical significance (Figure S2). Across all 3 signatures, higher signature z-scores were not associated with longer PFS or OS (Figure S3).

## 3. Discussion

Ramucirumab demonstrated antitumor activity and a favorable safety profile, which led to its approval in several indications, including second-line advanced G/GEJ cancer as single agent and in combination with paclitaxel [21,22]. Pembrolizumab also demonstrated antitumor activity and tolerable safety profile across different tumor types, and is currently approved for advanced G/GEJ PD-L1 CPS ≥ 1 (per 22C3 assay) patients who have received ≥ 2 lines of chemotherapy [16]. However, both agents failed to demonstrate survival benefit over chemotherapy in the first-line setting [8,14]. 

Here we report the results from the JVDF phase 1a/b trial, where a first-line advanced/metastatic G/GEJ adenocarcinoma cohort of 28 patients was treated with ramucirumab and pembrolizumab. This combination was well tolerated, and the overall safety profile was similar to that reported for 92 patients in late line JVDF G/GEJ, non-small cell lung cancer and urothelial carcinomas cohorts [26]. The frequency of TRAEs was higher compared with pembrolizumab monotherapy [14,16,20] or other JVDF [26] cohorts, but there was not more treatment-related discontinuations, with only 2 patients discontinuing due to a TRAE. No grade 4 treatment-related AEs or deaths were reported. All AEs were manageable with supportive care alone and/or with dose delays, without a substantial reduction in the planned dose intensity for either study drug.

In the KEYNOTE-062 phase 3 pembrolizumab monotherapy study of first-line patients with PD-L1-positive tumors (CPS ≥ 1, per 22C3 assay), most patients rapidly progressed and sequenced into second- or third-line treatment, with a median PFS of 2.0 months compared to 6.4 months for the chemotherapy arm [14]. PFS for patients with CPS ≥ 10 was similar (2.9 months) [14]. Although response rate and median OS (14.5% and 10.6 months, respectively, in KEYNOTE-062) for PD-L1 CPS ≥ 1 patients [14] was lower than rates initially reported in the first-line monotherapy arm phase 2 study (KEYNOTE-059: ORR, 25.8%; OS, 20.7 months) [27], pembrolizumab alone was noninferior to chemotherapy for OS as first-line treatment for patients with CPS ≥ 1 advanced G/GEJ cancer. In our JVDF study, we observed a durable ORR of 25% in the total PD-L1 unselected population (median DOR was yet to be reached at the time of data cutoff, with lower limit CI of 9.7 months), with a higher response rate in patients with PD-L1-positive CPS ≥ 1 (32%) versus PD-L1-negative (17%) tumors, as well as longer median PFS (8.6 month vs 4.3 months) and median OS (17.3 months vs 11.3 months). Although the prevalence of patients with PD-L1-negative tumors was low (21%), these results suggest an association between PD-L1-positive expression on tumor and immune cells measured by the investigational 22C3 assay and increased antitumor activity, which has also been observed in the second- and third-line G/GEJ cohort in JVDF [26], and in the pretreated G/GEJ cohort of KEYNOTE-059 [16]. Moreover, the 12-month OS rate of 66.7% with ramucirumab plus pembrolizumab compared favorably to the 47% observed in KEYNOTE-062 with pembrolizumab alone for CPS ≥ 1 tumors [14]. For CPS ≥ 10 tumors, the 12-month OS rate was 80% in this study compared to 57% in KEYNOTE-062 [14]. We observed a 12-month PFS rate of 35.5% for PD-L1 CPS ≥ 1 tumors compared to 14% with pembrolizumab alone in KEYNOTE-062 [14]. Recognizing caveats of cross-trial comparisons and a small sample size within our study, our results suggest there could be an enhanced effect when combining ramucirumab with pembrolizumab in the first-line setting.

The combination of anti-VEGF/VEGFR2 signaling inhibitors with checkpoint inhibitors has shown efficacy over standard-of-care treatment in multiple tumor types, including lung cancer [28], renal cell carcinoma [29,30,31], and hepatocellular carcinoma [32]. Therefore, the results of our study and others support modulating the tumor microenvironment with a combination of VEGF-pathway agents and PD-1/PD-L1-targeting agents. Ramucirumab and other anti-VEGF-VEGFR2 agents may modulate the tumor microenvironment to be more immunosupportive or inflammatory, and increase the efficacy of checkpoint inhibitors via several possible mechanisms, including increasing the trafficking and infiltration of CD8+ T cells into the tumor, increasing the expression of major histocompatibility complex-1 and PD-L1, and reducing the frequency of immunosuppressive regulatory T cells markers [23,24,25].

A pre-existing inflamed tumor microenvironment characterized by active IFNγ signaling and activated T cells was previously described to predict for clinical response to anti-PD-1/PD-L1 therapies in various tumor types [18,19,33]. To better understand the clinical activity of ramucirumab and pembrolizumab, we investigated the expression of 3 immune-related gene signatures and possible relation with clinical outcomes after ramucirumab and pembrolizumab treatment. We did not see a significant association of high T-effector and T cell-inflamed signature median scores with benefit after treatment with ramucirumab in combination with pembrolizumab in our subset of first line metastatic G/GEJ cancer patients that had available gene expression data, consistent with the recent analysis in the combined G/GEJ cohorts [34]. Limitations of this exploratory analysis included the small number of tumor samples, and lack of tissue tumor mutational burden status and of post-treatment samples.

While the combination of ramucirumab with pembrolizumab has produced durable antitumor activity, the small size of this G/GEJ first-line cohort and the single-treatment arm design of our study do not allow for efficacy conclusions, and further randomized controlled studies are required. Translational studies are also needed to better understand the biology of the tumor microenvironment and the immunomodulatory role of ramucirumab when combined with a checkpoint inhibitor. 

## 4. Materials and Methods

### 4.1. Pateints

Cohort A2 included patients ≥ 18 years old with untreated locally advanced or metastatic unresectable G/GEJ cancer who were ineligible for or refused standard chemotherapy for first-line treatment (patients whose disease had progressed after > 12 months following the last dose of systemic treatment in the adjuvant/neoadjuvant setting were eligible), and had measurable disease, as determined by the study site team, per Response Evaluation Criteria in Solid Tumors (RECIST) version 1.1 [35]. Other criteria were an Eastern Cooperative Oncology Group performance status of 0 or 1, a newly obtained tumor biopsy prior to enrollment (archived samples were allowed in certain circumstances), and adequate organ function.

Exclusion criteria included squamous cell or undifferentiated G/GEJ cancer, previous systemic chemotherapy for advanced or metastatic disease, and a HER-2-positive or -unknown status. More detailed inclusion and exclusion criteria have been previously published [26]. 

### 4.2. Study Design and Treatment

This open-label, single arm, non-randomized phase 1a/b study-commenced based on an acceptable number of dose-limiting toxicities (≤1) in the observation period of phase 1a as previously reported [26]. A protocol amendment in February 2016 [26] included 3 new phase 1b expansion cohorts, including Cohort A2, the first-line advanced Gastric/GEJ cohort reported here. Patients received ramucirumab 8 mg/kg intravenously on days 1 and 8 plus pembrolizumab 200 mg intravenously on day 1 every 3 weeks (1 cycle). Treatments were continued for up to 35 cycles, until confirmed progressive disease or discontinuation for any other reason. A protocol amendment in February 2019 allowed patients to continue ramucirumab treatment after cycle 35. Dose delays and discontinuation of either study drug were allowed if deemed necessary by the principal investigator to manage adverse events (AEs), or for other reasons. Dose reductions were allowed for ramucirumab only (pembrolizumab could be withheld).

This study was conducted in accordance with the International Conference on Harmonization Good Clinical Practice guidelines, the Declaration of Helsinki, applicable local regulations, and was approved by each institution’s ethical review board and registered at Clinicaltrials.gov (registration number NCT02443324). Patients provided written informed consent before study entry. 

### 4.3. Outcomes and Assessments

The primary endpoint, assessed by AE incidence, was safety and tolerability of ramucirumab plus pembrolizumab. Secondary endpoints included PFS, OS, objective response rate (ORR), disease control rate (DCR), time to response (TTR), duration of response (DOR), and pharmacokinetics of ramucirumab when administered with pembrolizumab. Definitions of efficacy endpoints are listed in Table S7. Exploratory endpoints included the association between biomarkers and clinical outcomes.

Safety was evaluated throughout the study and for 30 days after treatment discontinuation. AEs were graded using the National Cancer Institute Common Terminology Criteria for Adverse Events, version 4.0, and judged by the investigator as related or unrelated to study treatment.

Tumor response was assessed radiographically by the investigator according to RECIST version 1.1 [35], with required investigator confirmation for partial or complete response (PR or CR). Responses were assessed every 6 weeks (±7 days) for the first 24 weeks and every 12 weeks (±7 days) thereafter. Study treatment could continue until confirmed progressive disease in a second scan performed approximately 4 weeks after initial progressive disease was detected, to account for the observation that some patients can have a transient tumor flare in the first few months after beginning immunotherapy, but with subsequent disease response. Once progression was confirmed, treatment was discontinued.

If the patient was alive at data cutoff for the OS analysis, or was lost to follow-up, OS was censored on the last date the patient was known to be alive.

Serum ramucirumab concentration was measured using a validated enzyme-linked immunosorbent assay method (Charles River Laboratories, Senneville, QC, Canada).

### 4.4. Biomarker Analysis

Pre-treatment formalin-fixed paraffin-embedded tumor biopsy samples were collected prior to treatment for the biomarker analysis described here. Microsatellite instability (MSI) status was determined using polymerase chain reaction (Promega MSI Analysis System; Promega Corp., Madison, WI, USA). Microsatellite high (MSI-H) was defined as alterations in ≥ 2 of 5 microsatellite markers; microsatellite stable (MSS) was defined as no alterations. 

PD-L1 expression was assessed by immunohistochemistry (IHC) with an investigational version of the PD-L1 IHC 22C3 pharmDx assay (Agilent, Carpinteria, CA, USA) using CPS which is the number of PD-L1 stained tumor and immune cells divided by the total number of tumor cells and multiplied by 100. PD-L1 positivity was defined as CPS ≥ 1. Additional exploratory analyses used a CPS cutoff ≥ 10, noting that CPS ≥ 10 was a subset of CPS ≥ 1. 

Patient tumor samples were profiled on the Nanostring nCounter PanCancer Immune Profiling panel using extracted RNA from 10 patients [36]. Adequate tumor tissue was not available for other patients. Raw gene expression data was normalized to internal controls followed by log2 transformation, and Z-scoring scaling across the 10 patients. Study parameters were visualized using the ComplexHeatmap package in R [37]. 

Three immune-related gene expression signatures were selected and adapted based on the genes present in the Nanostring panel for in-depth analysis: T cell-effector, T cell-inflamed/tumor inflammation signature (TIS), and T cell-inflamed signature (modified Gajewski). These gene signatures represent a means of quantifying the level of tumor microenvironment inflammation as characterized by active IFNγ signaling, cytotoxic effector molecules, antigen presentation, and T cell recruitment [33]. Patient signature scores were calculated as the average of the constituent genes as previously described [17]. 

### 4.5. Statistical Analysis

The data cutoff for all analyses was 21 April 2019. The sample size was selected to allow adequate assessment of safety and efficacy at the recommended doses for ramucirumab and pembrolizumab. The null hypothesis was based on the assumption that the ORR would be no greater than 30% to 35% and the target treatment effect of the combination treatment on ORR would be greater than 45% to 55%. Based on these assumptions, a sample size of *n* = 25 provided statistical power of approximately 65% to 90%, with a 1-sided 0.20 significance level. All patients who received ≥ 1 dose of ramucirumab or pembrolizumab were evaluated for safety and efficacy. Time-to-event variables were estimated with the Kaplan-Meier method. Safety and efficacy data were analyzed using SAS version 9.4; exploratory analyses were analyzed with R versions 3.5 and 3.6.0. 

Pearson correlations assessed the relationship between PD-L1 gene expression (CD274) and immune related signatures. The association between T cell signatures and ORR was evaluated with T-tests. Cox proportional hazards model assessed the association between T cell signatures and PFS/OS.

### 4.6. Nanostring Methods

RNA count data (.RCC files) were normalized using the geometric mean of the positive controls and housekeeping genes using in-house implementation of the NanoStringNorm R package [36]. The lower limit of detection (LLOD) for counts was set at the maximum count of the background (i.e., negative) controls; 114 genes with normalized counts less than the LLOD were flagged as low-expressing genes and removed from downstream analyses and 616 genes passed quality control and were further investigated.

Genes constituting the T cell-effector signature included: CD8A, CXCL9, CXCL10, EOMES, GZMA, GZMB, IFNG, PRF1, and TBX21 [18]. Genes constituting the T cell-inflamed (TIS) signature included: CCL5, CD27, CD274, CD276, CD8A, CMKLR1, CXCL9, CXCR6, HLA-DQA1, HLA-E, IDO1, LAG3, PDCD1LG2, PSMB10, STAT1, and TIGIT (HLA-DRB5 and NKG7 were not present on the Nanostring Immune panel, and therefore could not be included) [17]. Genes constituting the T cell-inflamed (modified Gajewski) signature included: CCL2, CCL3, CCL4, CD8A, CXCL10, CXCL9, GZMK, HLA-DMA, HLA-DMB, HLA-DOB, ICOS, and IRF1 (HLA-DOA was not present on the Nanostring Immune panel, and therefore could not be included) [38]. 

### 4.7. Data Availability

Lilly provides access to all individual participant data collected during the trial, after anonymization, with the exception of pharmacokinetic or genetic data. Data are available to request 6 months after the indication studied has been approved in the US and EU and after primary publication acceptance, whichever is later. No expiration date of data requests is currently set once data are made available. Access is provided after a proposal has been approved by an independent review committee identified for this purpose and after receipt of a signed data sharing agreement. Data and documents, including the study protocol, statistical analysis plan, clinical study report, blank or annotated case report forms, will be provided in a secure data sharing environment. Requests for data should be submitted at www.vivli.org.

## 5. Conclusions

In conclusion, in treatment-naïve advanced or metastatic G/GEJ, the safety profile of ramucirumab plus pembrolizumab was consistent with monotherapy treatment for each drug, with no unexpected toxicities. Acknowledging limitations of sample size, efficacy results of the combination were encouraging. ORR and DCR were similar to first-line chemotherapy, but more durable than expected, and survival outcomes were numerically superior to first-line chemotherapy, especially among patients with PD-L1-positive tumors.

## Figures and Tables

**Table 1:** Baseline demographic and clinical characteristics.

*n* (%), Unless Otherwise Indicated	Ramucirumab + Pembrolizumab *N* = 28
Sex	
Female	7 (25)
Male	21 (75)
Age	
Median years (range)	63 (31–83)
≤65 years	14 (50)
Race	
Black or African American	1 (4)
White	17 (61)
Unknown or not reported	10 (36)
Ethnicity	
Hispanic or Latino	3 (11)
Not Hispanic or Latino	16 (57)
Unknown or not reported	9 (32)
ECOG PS	
0	16 (57)
1	12 (43)
Disease stage	
Metastatic	27 (96)
Non-metastatic	1 (4)
Histopathological diagnosis	
Well differentiated	2 (7)
Moderately differentiated	11 (39)
Poorly differentiated	13 (46)
Unable to determine	2 (7)
Tumor location	
Gastric	17 (61)
Gastroesophageal junction	11 (39)
HER2 negative	28 (100)
PD-L1 status	
Positive ^1^	19 (68)
Negative	6 (21)
Not reported ^2^	3 (11)
Microsatellite instability	
High	2 (7)
Stable	15 (54)
Not available	11 (39)
Prior surgery ^3^	11 (39)
Prior radiotherapy	6 (21)
Prior systemic therapy ^4^	
≥1 prior systemic therapy	7 (25)
Adjuvant	4 (14)
Neoadjuvant	4 (14)

^1^ PD-L1-positive status is defined as combined positive score ≥ 1. ^2^ Tissue was inadequate or failed testing. ^3^ Three patients had gastrectomy. ^4^ Two patients received >1 systemic therapy in the neoadjuvant and/or adjuvant setting. Abbreviations: ECOG PS, Eastern Cooperative Oncology Group performance status; HER2, human epidermal growth factor receptor 2; *n*, number of patients in a given sample; *N*, number of patients in overall population; PD-L1, programmed death-ligand 1.

**Table 2:** Summary of treatment-related adverse events and treatment-emergent adverse events of special interest ^1^, irrespective of relatedness occurring in ≥ 5% of the total patient population.

*N* = 28 *n* (%)	Grade 1 or Grade 2	Grade 3 ^2^
TRAEs by preferred term		
Fatigue ^3^	11 (39)	0
Headache	6 (21)	0
Rash ^4^	6 (21)	1 (4)
Epistaxis	5 (18)	0
Nausea	5 (18)	0
Proteinuria	5 (18)	0
Stomatitis	5 (18)	0
Decreased appetite	4 (14)	0
Hypertension	4 (14)	4 (14)
Anemia	3 (11)	0
Diarrhea	3 (11)	2 (7)
Peripheral edema	3 (11)	0
ALT increased	2 (7)	3 (11)
AST increased	2 (7)	3 (11)
Chills	2 (7)	0
Colitis	2 (7)	0
Dyspnea	2 (7)	0
Hematuria	2 (7)	0
Influenza-like illness	2 (7)	0
Myalgia	2 (7)	0
Decreased weight	1 (4)	1 (4)
Gastrointestinal hemorrhage	0	2 (7)
AEs of special interest for ramucirumab		
Epistaxis	6 (21)	0
Hematuria	3 (11)	0
Gastrointestinal hemorrhage	0	2 (7)
Hypertension	4 (14)	7 (25)
Proteinuria	5 (18)	1 (4)
Deep vein thrombosis	2 (7)	1 (4)
Angina pectoris	2 (7)	1 (4)
Blood creatinine increased	1 (4)	1 (4)
Immune-related AEs for pembrolizumab		
Diarrhea	9 (32)	2 (7)
Colitis	2 (7)	0
Rash maculo-papular	2 (7)	1 (4)
Rash	2 (7)	0
ALT increase	2 (7)	3 (11)
AST increased	2 (7)	3 (11)
Acute kidney injury	1 (4)	1 (4)
Blood creatinine increased	1 (4)	1 (4)
Pneumonitis	2 (7)	0

^1^ TRAEs, AEs of special interest for ramucirumab, and immune-related AEs for pembrolizumab in ≥ 5% of patients are presented. AEs are presented at grade 1-2 and grade 3, according to preferred term, unless otherwise indicated, and presented as consolidated events. ^2^ No grade 4 or 5 events were reported. ^3^ Consolidated events were: fatigue, where the terms fatigue and asthenia were combined; and rash, where the terms rash maculo-papular, rash, dermatitis acneiform, and rash pruritic were combined. Abbreviations: AE, adverse event; ALT, alanine aminotransferase; AST, aspartate aminotransferase; *n*, number of patients in a given sample; *N*, number of patients in overall population; TRAE, treatment-related adverse event.

**Table 3:** Response, as confirmed per RECIST, and survival.

	All ^1^ *N* = 28	PD-L1-Negative (CPS < 1) *N* = 6	PD-L1-Positive (CPS ≥ 1) *N* = 19	CPS ≥ 10 ^2^ *N* = 10
Best overall response, *n* (%)				
Complete response	1 (4)	0	1 (5)	0
Partial response	6 (21)	1 (17)	5 (26)	4 (40)
Stable disease	12 (43)	3 (50)	7 (37)	4 (40)
Progressive disease	7 (25)	1 (17)	6 (32)	2 (20)
Not evaluable	2 (7)	1 (17)	0	0
Objective response rate, % (95% CI)	25 (10.7–44.9)	17 (0.4–64.1)	32 (12.6–56.6)	40 (12.2–73.8)
Disease control rate, % (95% CI)	68 (47.6–84.1)	67 (22.3–95.7)	68 (43.4–87.4)	80 (44.4–97.5)
Time to response, median months (95% CI)	2.7 (1.3–2.8)	2.8 (NC)	2.1 (1.3–9.8)	1.4 (1.3–2.8)
Duration of response, median months (95% CI)	NR (9.7–NC)	NR (NC)	NR (9.7–NC)	NR (9.7–NC)
Duration of stable disease, median months (95% CI)	5.6 (3.9–12.3)	5.1 (4.3–5.8)	8.6 (4.1–13.5)	5.0 (4.1–13.5)
Progression-free survival				
Number of events	20	4	14	8
Median duration, months (95% CI)	5.6 (2.7–11.5)	4.3 (2.4–NR)	8.6 (1.5–13.5)	8.3 (1.2–13.5)
6-month rate, % (95% CI)	42.9 (23.9–60.6)	20.8 (0.9–59.5)	52.6 (28.7–71.9)	50.0 (18.4–75.3)
12-month rate, % (95% CI)	30.3 (14.0–48.4)	20.8 (0.9–59.5)	35.5 (15.2–56.6)	30.0 (7.1–57.8)
18-month rate, % (95% CI)	20.8 (7.3–38.9)	20.8 (0.9–59.5)	22.2 (6.3–44.0)	20.0 (3.1–47.5)
Overall survival				
Number of events	17	3	11	5
Median duration, months (95% CI)	14.6 (5.4–27.7)	11.3 (2.4–NR)	17.3 (8.6–NR)	24.7 (5.4–NR)
6-month rate, % (95% CI)	69.4 (48.0–83.4)	62.5 (14.2–89.3)	77.8 (51.1–91.0)	90.0 (47.3–98.5)
12-month rate, % (95% CI)	54.0 (33.4–70.7)	41.7 (5.6–76.7)	66.7 (40.4–83.4)	80.0 (40.9–94.6)
18-month rate, % (95% CI)	40.9 (21.7–59.2)	41.7 (5.6–76.7)	48.1 (23.6–69.0)	57.1 (21.7–81.5)

^1^ Includes patients with unknown PD-L1 status. ^2^ The CPS ≥ 10 group is a subset of the CPS ≥ 1 group. Abbreviations: CI, confidence interval; CPS, combined positive score; *n*, number of patients in a given sample; *N*, number of patients in overall population; NC, not calculable; NR, not reached; PD-L1, programmed death-ligand 1; RECIST, Response Evaluation Criteria in Solid Tumors.

## Supplementary Materials

The following are available online at https://www.mdpi.com/2072-6694/12/10/2985/s1, Figure S1: CONSORT diagram in the treatment-naïve, advanced gastric/gastroesophageal junction (G/GEJ) cancer Cohort A2. Figure S2: Biomarker immune profiling analysis in 10 1L G/GEJ patients, Figure S3: Association between the 3 gene signatures, Table S1: Dose administration, Table S2: Treatment-emergent adverse events, Table S3: Treatment-related serious adverse events, Table S4: Post-discontinuation treatment, Table S5: Summary of ramucirumab trough concentrations (pre-dose) for patients treated with 8 mg/kg of ramucirumab intravenously on days 1 and 8 Q3W in combination with pembrolizumab, Table S6: Summary of ramucirumab peak (1 h post-infusion) concentrations for patients treated with 8 mg/kg of ramucirumab intravenously on days 1 and 8 Q3W in combination with pembrolizumab, Table S7: Efficacy endpoints definition.
