# Supplementary material for: Ramucirumab in Combination with Pembrolizumab in Treatment-Naïve Advanced Gastric or GEJ Adenocarcinoma: Safety and Antitumor Activity from the Phase 1a/b JVDF Trial
Source: Cancers (Basel). 2020 Oct 15;12(10):2985. doi: 10.3390/cancers12102985 (PMC7602637; doi:10.3390/cancers12102985)
Supplement: Supplementary file 1 [file cancers-12-02985-s001.pdf]

cancers

MDPI

Article

Ramucirumab in Combination with Pembrolizumab in Treatment-Naïve Advanced Gastric or GEJ Adenocarcinoma: Safety and Antitumor Activity from the Phase 1a/b JVDF Trial

Ian Chau [1,*], Nicolas Penel [2], Andres O. Soriano [3], Hendrik-Tobias Arkenau [4], Jennifer Cultrera [5], Rafael Santana-Davila [6], Emiliano Calvo [7], Christophe Le Tourneau [8], Lars Zender [9], Johanna C. Bendell [10], Gu Mi [11], Ling Gao [11], Samuel Clark McNeely [11], Joana M. Oliveira [12], David Ferry [12], Roy S. Herbst [13] and Charles S. Fuchs [13,14]

1 Gastrointestinal Units, Royal Marsden Hospital, London SM2 5PT, UK
2 General Oncology Department, Medical Oncology Department, Oscar Lambret Cancer Center, Lille 59020, France; n-penel@o-lambret.fr
3 Sarah Cannon Research Institute/Florida Cancer Specialists, Englewood, FL 34223, USA; asoriano@flcancer.com
4 Sarah Cannon Research Institute United Kingdom and University College London, London, W1G 6AD, UK; Tobias.Arkenau@HCAHealthcare.co.uk
5 Sarah Cannon Research Institute/Florida Cancer Specialists Leesburg South, Leesburg, FL 34748, USA; JCultrera@flcancer.com
6 Department of Medicine, University of Washington Medicine, Seattle Cancer Care Alliance, Seattle, WA 98109, USA; RSantana@seattlecca.org
7 Early Phase Clinical Drug Development Program, START Madrid-CIOCC, Centro Integral Oncológico Clara Campal, Madrid 28050, Spain; ECalvo@hmhospitales.com
8 Department of Drug Development and Innovation, Institut Curie, Paris 75005, France; Christophe.LeTourneau@curie.fr
9 Department of Internal Medicine VIII, University Hospital Tübingen, Tübingen 72076, Germany; Lars.Zender@med.uni-tuebingen.de
10 Sarah Cannon Research Institute/Tennessee Oncology, Nashville, TN 37203, USA; JBendell@tnonc.com
11 Eli Lilly and Company, Indianapolis, IN 46285, USA; Mi_Gu@lilly.com (G.M.); LingGao418@gmail.com (L.G.); McNeely_Samuel_Clark@lilly.com (S.C.M.)
12 Eli Lilly and Company, New York, NY 10016, USA; Oliveira_Joana@lilly.com (J.M.O.); Ferry_David@lilly.com (D.F.)
13 Yale Cancer Center, New Haven, CT 06520, USA; Roy.Herbst@yale.edu (R.S.H.); Charles.Fuchs@yale.edu (C.S.F.)
14 Smilow Cancer Hospital, New Haven, CT 06473, USA
* Correspondence: Ian.Chau@rmh.nhs.uk; Tel.: +44-20-8915-6196

Received: 28 August 2020; Accepted: 13 October 2020; Published: date

Supplementary

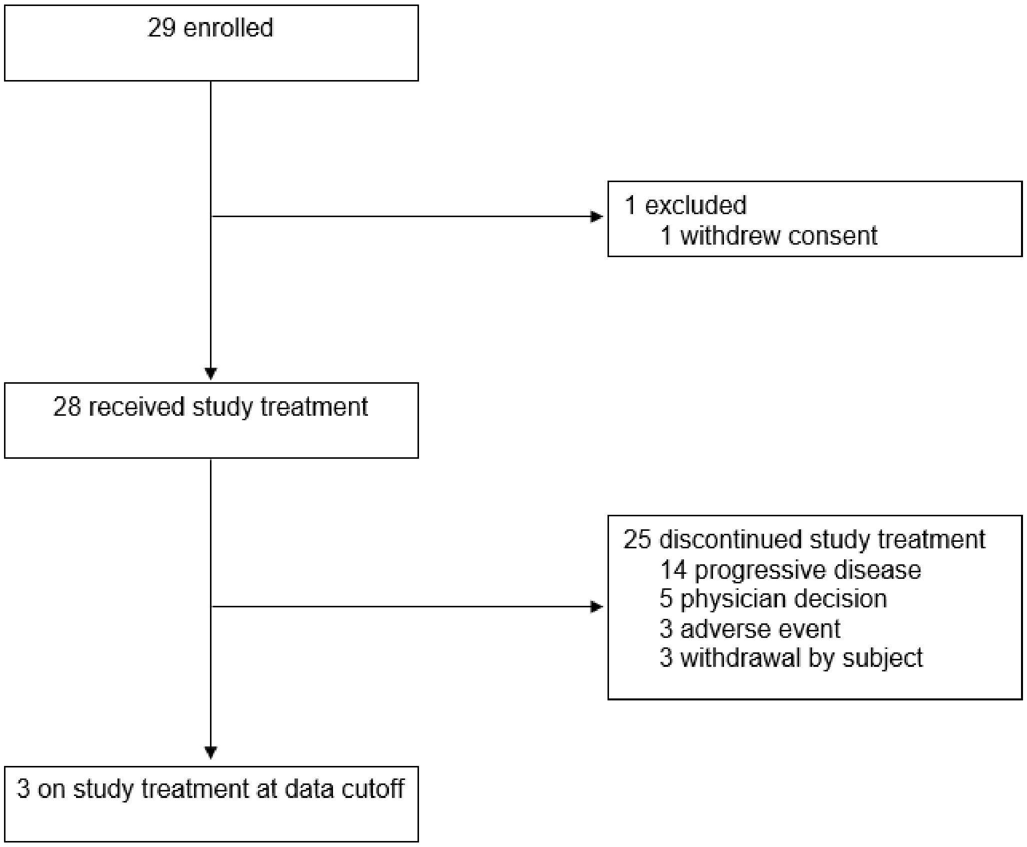

Figure S1. CONSORT diagram in the treatment-naïve, advanced gastric/gastroesophageal junction (G/GEJ) cancer Cohort A2.

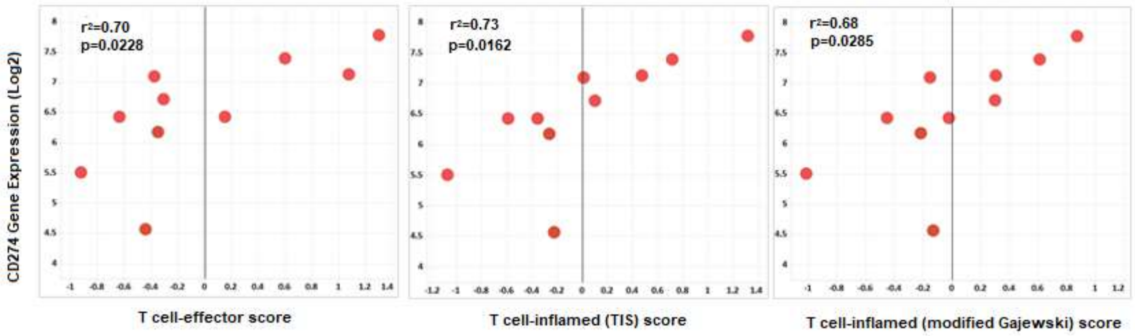

(a)

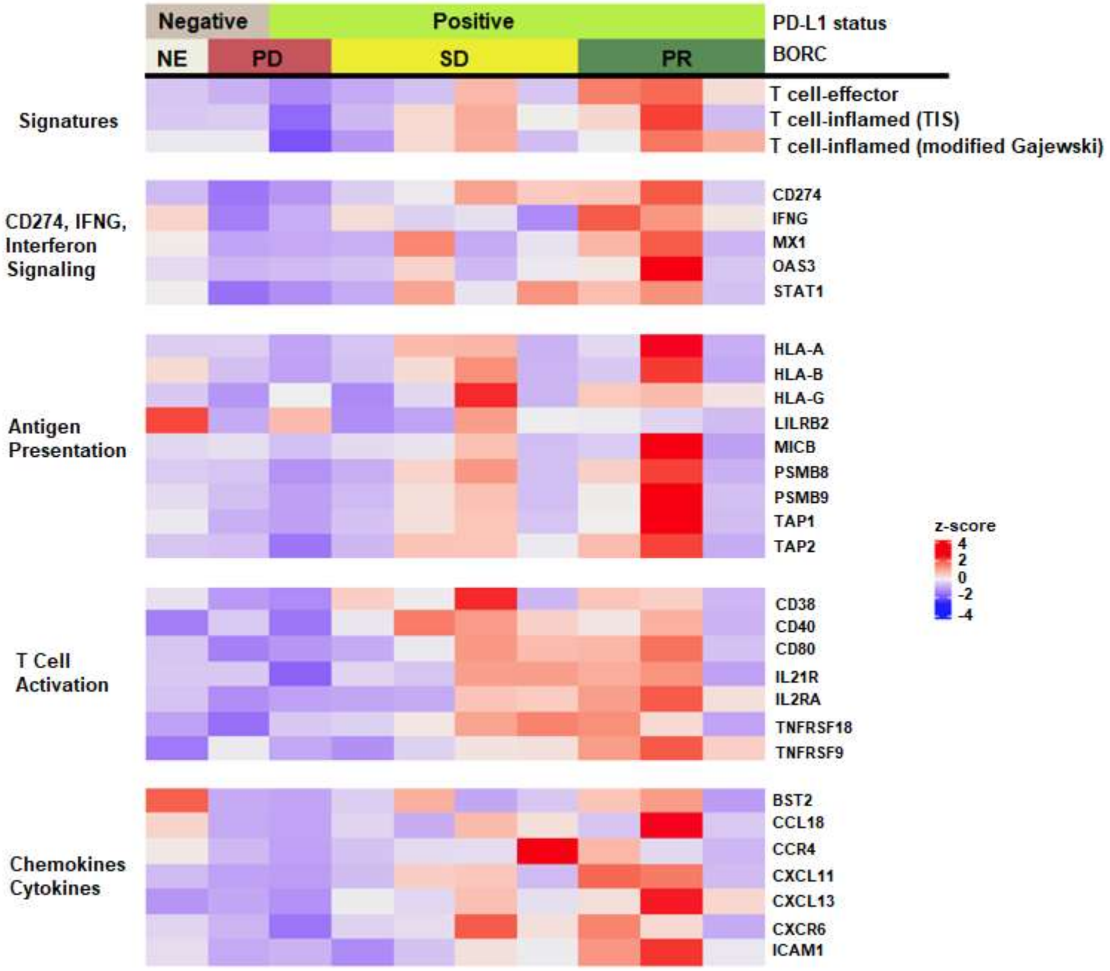
Negative
Positive
PD-L1 status
NE
PD
SD
PR
BORC
Signatures
T cell-effector
T cell-inflamed (TIS)
T cell-inflamed (modified Gajewski)
CD274, IFNG, Interferon Signaling
CD274
IFNG
MX1
OAS3
STAT1
Antigen Presentation
HLA-A
HLA-B
HLA-G
LILRB2
MICB
PSMB8
PSMB9
TAP1
TAP2
T Cell Activation
CD38
CD40
CD80
IL21R
IL2RA
TNFRSF18
TNFRSF9
Chemokines Cytokines
BST2
CCL18
CCR4
CXCL11
CXCL13
CXCR6
ICAM1
z-score
4
2
0
-2
-4

(b)

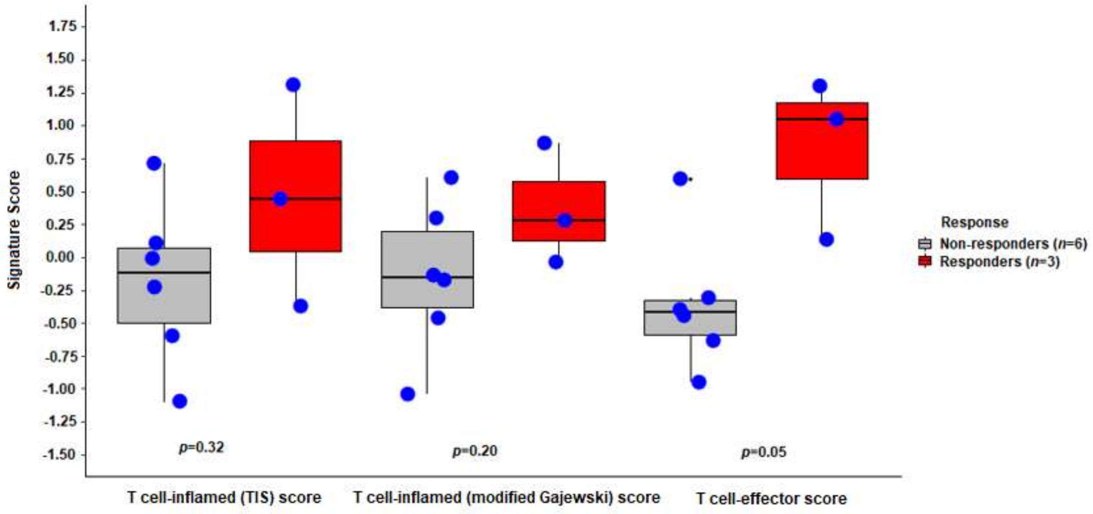
Signature Score
1.75
1.50
1.25
1.00
0.75
0.50
0.25
0.00
-0.25
-0.50
-0.75
-1.00
-1.25
-1.50
p=0.32
p=0.20
p=0.05
T cell-inflamed (TIS) score
T cell-inflamed (modified Gajewski) score
T cell-effector score
Response
Non-responders (n=6)
Responders (n=3)

(c)

Figure S2. Biomarker immune profiling analysis in 10 1L G/GEJ patients (**a**) Pearson correlations of CD274 (PD-L1 gene) expression with gene signatures scores, (**b**) expression profiling of the 3 gene signatures and other genes of interest by PD-L1 protein expression and BORC, and (**c**) boxplot showing the relationship between the 3 gene signature scores and objective response by RECIST. The horizontal line represents the median. Responder, complete or partial response; nonresponder, stable disease or progressive disease. Not evaluable patients (*n*=1) excluded from this analysis. Abbreviations: BORC, best overall response (confirmed); *n*, number in sample; NE, not evaluable; PD, progressive disease; PR, partial response; SD, stable disease.

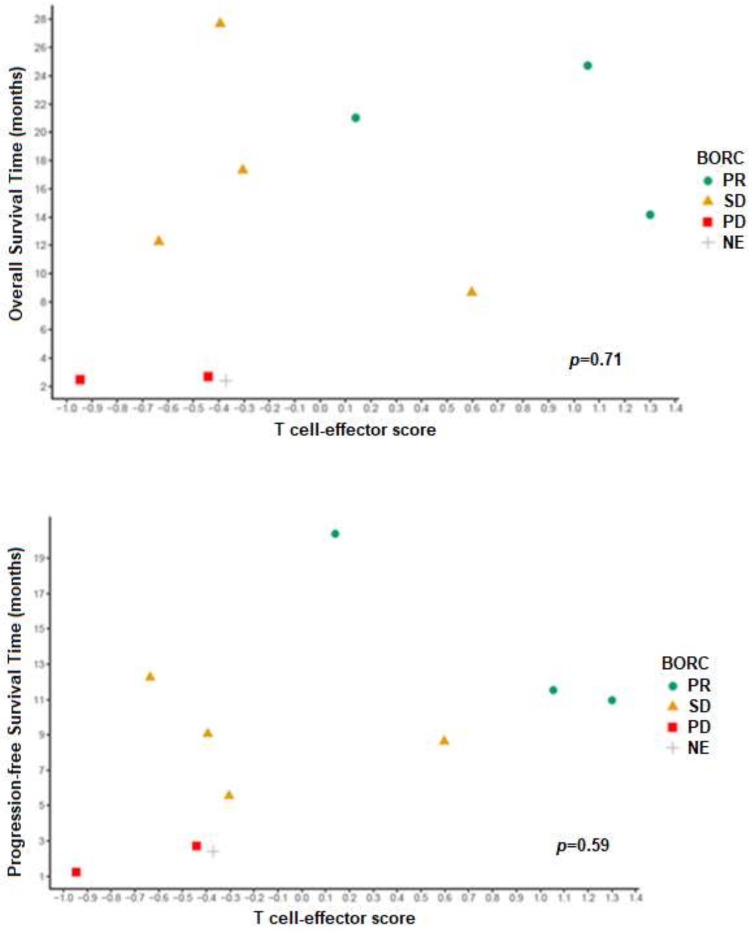

(a)

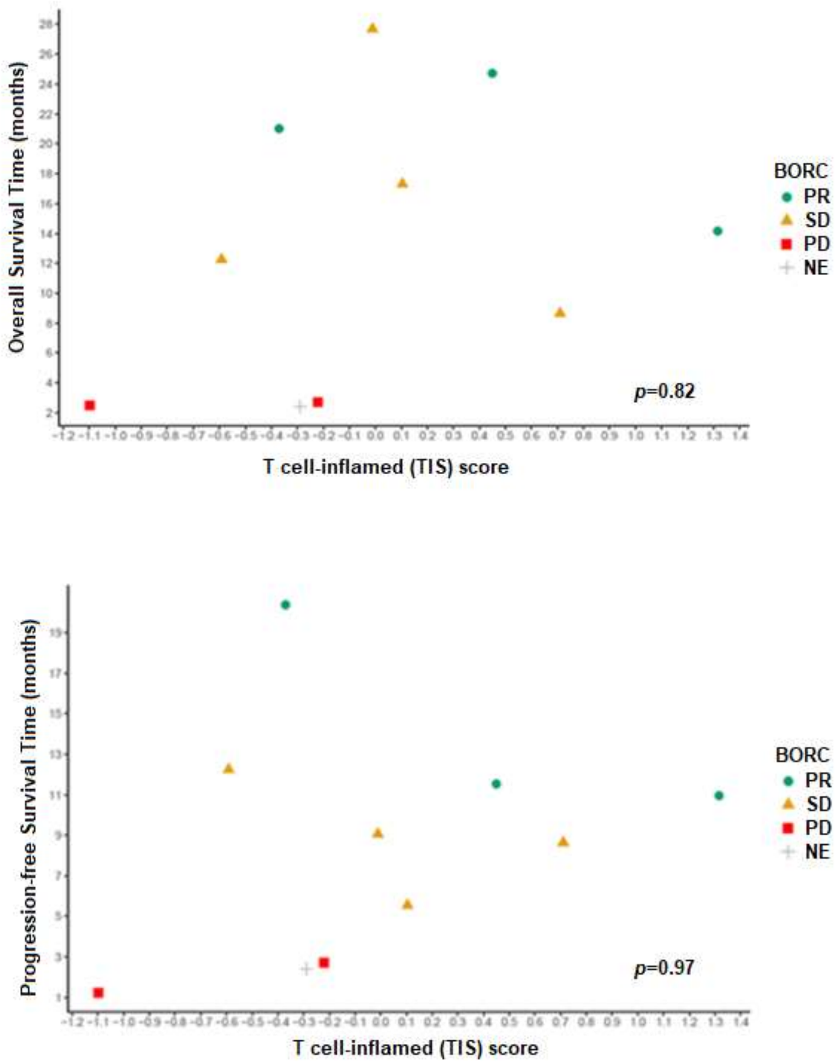
Overall Survival Time (months)
T cell-inflamed (TIS) score
BORC
PR
SD
PD
NE
p=0.82
Progression-free Survival Time (months)
T cell-inflamed (TIS) score
BORC
PR
SD
PD
NE
p=0.97

(b)

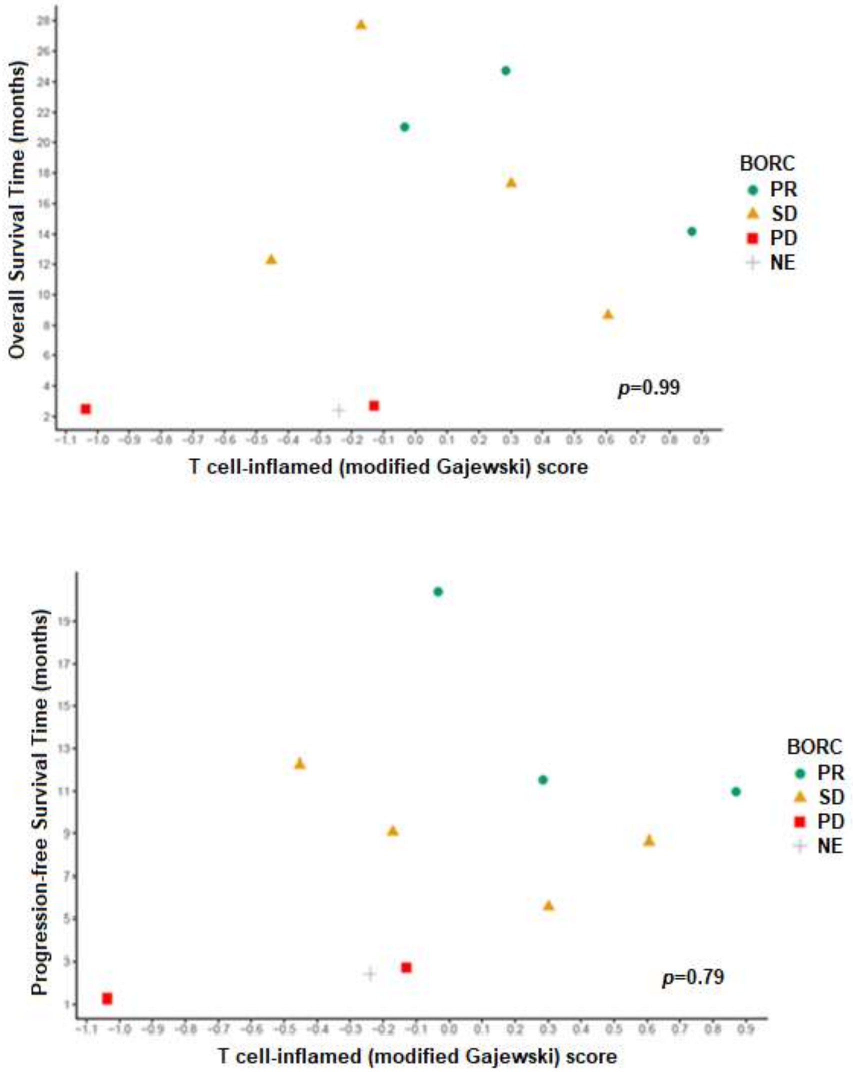

(c)

Figure S3. Association between the 3 gene signatures (**a**) T cell-effector score, (**b**) T cell-inflamed (TIS) score, and (**c**) T cell-inflamed (modified Gajewski) score, with progression-free survival and overall survival after ramucirumab and pembrolizumab treatment for 10 patients.
Abbreviations: NE, not evaluable; PD, progressive disease; PR, partial response; SD, stable disease.

Table S1. Dose administration.

	**Ramucirumab + pembrolizumab** *N* = 28	
	**Ramucirumab**	**Pembrolizumab**
Duration of therapy, median months (IQR)	4.5 (2.1 – 9.7)	4.5 (2.1 – 10.1)
Number of cycles received, median (IQR)	7 (3 – 13)	7 (3 – 14)
Relative dose intensity, median % (range)	94 (65 – 103)	98 (66 – 101)
Dose reductions, *n* (%)	2 (7)	Not applicable
Dose delays, *n* (%)	19 (68)	11 (39)
Treatment interrupted, *n* (%)	1 (4)	0

Abbreviations: IQR, interquartile range; *n*, number in sample; *N*, number of patients in overall population.

Table S2. Treatment-emergent adverse events[1].

***n* (%)**	**Ramucirumab + pembrolizumab *N* = 28**
TEAEs	
≥1 TEAE	28 (100)
Fatigue	19 (68)
Nausea	12 (43)
Diarrhea	11 (39)
Hypertension	11 (39)
Decreased appetite	8 (29)
Dyspnoea	8 (29)
Headache	8 (29)
Peripheral edema	8 (29)
Vomiting	8 (29)
Abdominal pain	6 (21)
Epistaxis	6 (21)
Proteinuria	6 (21)
Abdominal pain upper	5 (18)
ALT increased	5 (18)
AST increased	5 (18)
Constipation	5 (18)
Dysphagia	5 (18)
Stomatitis	5 (18)
Urinary tract infection	5 (18)

[1] Any grade TEAEs in ≥ 15% of patients are presented.Abbreviations: ALT, alanine aminotransferase; AST, aspartate aminotransferase; *n*, number in sample; *N*, number of patients in overall population; TEAE, treatment-emergent adverse event.

Table S3. Treatment-related serious adverse events[1].

***n* (%)**	**Ramucirumab + pembrolizumab *N* = 28**
SAEs	
≥1 SAE	11 (39)
Colitis	2 (7)
Diarrhea	2 (7)
Gastrointestinal hemorrhage	2 (7)

[1] SAEs in ≥5% of patients by preferred term are presented. Abbreviations: *n*, number in sample; *N*, number of patients in overall population; SAE, serious adverse event.

Table S4. Post-discontinuation treatment[1].

***n* (%)**	**Ramucirumab + pembrolizumab *N* = 28**
Patients who received ≥1 PDT	13 (47)
Patients who underwent ≥1 surgery	3 (11)
Stomach and lymph node resection	1 (4)
Other procedures	2 (7)
Any systemic anticancer therapy	
5-fluorouracil	8 (29)
Oxaliplatin	8 (29)
Cisplatin	3 (11)
Leucovorin	3 (11)
Capecitabine	2 (7)
Irinotecan	2 (7)
Carboplatin	1 (4)
Epirubicin	1 (4)
Paclitaxel	1 (4)

[1] Patients may have had more than one therapy.Abbreviations: *n*, number in sample; *N*, number of patients in overall population; PDT, post-discontinuation treatment.

Table S5. Summary of ramucirumab trough concentrations (pre-dose) for patients treated with 8 mg/kg of ramucirumab intravenously on days 1 and 8 Q3W in combination with pembrolizumab.

	**Trough serum concentrations (μg/mL)**				
**Dose**	**3**	**5**	**7**	**9**	**13**
Week	3	6	9	12	18
n_{PK}	22	18	13	10	7
Min	24	31	51	45	46
Max	71	114	118	137	151
Geo mean[1]	45	61	79	80	88
Geo CV%[1]	32	38	31	38	48

[1] Not calculated when n_{PK} <3. Abbreviations: CV%, percentage coefficient of variation; Geo, geometric; Max, maximum; Min, minimum; n_{PK}, number of pharmacokinetic observations included in calculation; Q3W, every 3 weeks.

Table S6. Summary of ramucirumab peak (1 hour post-infusion) concentrations for patients treated with 8 mg/kg of ramucirumab intravenously on days 1 and 8 Q3W in combination with pembrolizumab.

	**Peak serum concentrations (μg/mL)**
Dose	1
Week	0
n_{PK}	24
Min	102
Max	285
Geo mean[1]	150
Geo CV%[1]	24

[1] Not calculated when n_{PK} <3.Abbreviations: CV%, percentage coefficient of variation; Geo, geometric; Max, maximum; Min, minimum; n_{PK}, number of pharmacokinetic observations included in calculation; Q3W, every 3 weeks.

Table S7. Efficacy endpoints definitions.

**Objective response rate**	**The proportion of treated patients who achieved a best overall response of CR or PR.**
Time to response	The time from the date of first study treatment until the first evidence of a confirmed CR or PR.
Duration of response	Measured from the date of first evidence of a confirmed CR or PR to the date of objective progression or the date of death due to any cause, whichever was earlier, only for patients with a confirmed CR or PR. If a responder was not known to have died or have objective progression at the data inclusion cutoff date, the duration of response was censored at the date of the last complete objective progression-free disease assessment.
Disease control rate	The proportion of treated patients who had a best overall response of CR, PR, or SD.
Progression-free survival	The time from the date of first study treatment until the date of the first observed radiographically documented PD or death due to any cause, whichever was earlier.
Overall survival	Determined from the date of first study treatment until death due to any cause.

Abbreviations: CR, complete response; PD, progressive disease; PR, partial response; SD, stable disease.
